# Levels, trends, and determinants of effectiveness on the hierarchical medical system in China: Data envelopment analysis and bootstrapping truncated regression analysis

**DOI:** 10.3389/fpubh.2022.921303

**Published:** 2022-09-20

**Authors:** Yuanxin Hou, Wenjuan Tao, Shufen Hou, Weimin Li

**Affiliations:** ^1^Institute of Hospital Management, West China Hospital, Sichuan University, Chengdu, China; ^2^Department of Critical Care Medicine, Chongqing University Three Gorges Hospital, Chongqing, China; ^3^President's Office, West China Hospital, Sichuan University, Chengdu, China

**Keywords:** hierarchical medical system, efficiency, total factor productivity, influencing factors, China

## Abstract

**Background:**

The hierarchical medical system (HMS) refers to the classification of treatment according to disease priorities based on severity and difficulty to promote the fairness of medical services for residents, which is regarded as the key to the success of medical reform in China.

**Methods:**

In the past decade of “New Medical Reform,” the efficiency of HMS, including secondary and tertiary hospitals and primary healthcare centers (PHCs), was measured horizontally and vertically by employing the combination of an output-oriented superefficiency slack-based model-data envelopment analysis (SE-SBM-DEA) model with the Malmquist total factor productivity index (MTFP). In the second stage, the overall technical efficiency (OTE) scores were regressed against a set of environmental characteristics and several managerial factors through bootstrapping truncated regression.

**Results:**

On average, the OTE score in tertiary hospitals was 0.93, which was higher than that in secondary hospitals and PHCs (0.9 and 0.92, respectively). In terms of trend, the OTE of tertiary hospitals declined at first and then increased. The opposite was true of secondary hospitals, in which the APC of the OTE was 10.82 and −3.11% in early and late 2012, respectively. The PHCs generally showed a fluctuating downward trend. In the aspects of productivity, all institutions showed a downturn by an annual average rate of 2.73, 0.51, and 2.70%, respectively. There was a significant negative relationship between the ratio of outpatients to inpatients and tertiary hospitals. Additionally, the medical technical personnel per 1,000 population negatively affected PHCs. In contrast, the GDP per capita had a significantly positive effect on tertiary hospitals, and the number of beds per 1,000 population positively influenced PHCs.

**Conclusion:**

The efficiency of medical institutions at various levels in HMS was unbalanced and took the form of an “inverted pyramid.” Multilateral factors influence the efficiency of HMS, and to address it, multi-intervention packages focusing on sinking high-quality medical resources and improving healthcare capacity, and guiding hierarchical medical practice should be adopted.

## Introduction

China has been dedicated to promoting a hierarchical medical system (HMS) to rationally modify the current healthcare structure with the goal to realize equal access to basic medical services for all, ultimately, achieving maximized use of medical resources. In the 1980s, the establishment of a three-level medical system composed of city, district, and street clinics was the prototype of HMS in China. In March 2009, the Chinese government formally launched a new round of healthcare reform, of which HMS was considered the “core” content (1, 2). In 2015, the State Council proposed a tiered healthcare delivery system toward an official implementation of HMS in China. By 2020, the HMS conforming to national conditions will be established (3). To achieve such a goal, a series of promotion strategies contributed to the implementation of HMS in hospitals nationwide, including training medical staff, doctors practicing in multiple locations, medical alliances (4), partner assistance, telemedicine (5), professional coalitions, family doctor contract services (6), and medical insurance (7).

Although the HMS in China has made some progress, there are still challenges, and more effective strategies are expected to be implemented in the future (8). For example, the structural questionnaire in Shandong found that self-initiated downward referral was less than one-third, and upward referral was easy but hard than downward referral (9). The nationally representative longitudinal data from 2012 to 2018 indicated that the residents' likelihood of choosing PHC services represented a decreasing trend (10). A practical survey in Jilin indicated that care users' first contact with PHCs flowed in an inverse U shape (11). In contrast, a study among patients showed that 2/5 patients bypassed PHCs to access care from higher-tier facilities (12). In fact, superior hospitals often form a certain degree of siphonic effect on subordinate hospitals due to the loose two-way referral and their technical advantages (13). An analysis of the hospital market in China after the 2009 healthcare reform showed that tertiary hospitals were still hard to access because of excessive patients and overlap in services with junior hospitals (14, 15), which usually indicated higher healthcare expenditures (16). The work-related accumulated fatigue among doctors in six provinces of China showed that high-level states were prevalent in tertiary hospitals (17), as well as idled medical resources in secondary hospitals and PHCs. To optimize the redistribution of medical resources and improve the efficiency of HMS, more needs to be done by the government (18).

Given the critical role that efficiency plays in improving the use of medical resources, understanding the level of efficiency and associated factors in HMS is an important research and policy question. Wang analyzed the efficiency of China's medical service resources under the background of HMS and found a large gap between provinces (19). At the provincial level, Wu examined the unbalanced implementation efficiency of PHCs in Fujian (20). According to the practical survey of Chongqing, Yang found that there were still significant spatial disparities in spatial accessibility to medical services by adopting HMS (21). Wang built a discrete choice model, which indicates that HMS led to an increase in patient satisfaction (22). Niu (23) and Li (24) systematically found that the HMS could gradually increase the total medical profit. However, there is no research evidence available to compare the efficiency of the three-level medical system in China. In this article, we focused on the comparison of effectiveness between secondary and tertiary hospitals and PHCs in HMS. Superefficiency slack-based model-data envelopment analysis (SE-SBM-DEA) and the DEA-based Malmquist total factor productivity index (MTFP, TFP) were combined to evaluate the static and dynamic efficiency of resource allocation in medical institutions at various levels from 2010 to 2019. A bootstrapping truncated regression model was used to perform a regression analysis on the influencing factors on technical effectiveness. In addition, the analytical framework has several technical advantages: first, the input and output of healthcare include multiple aspects, which is exactly the advantage of DEA for calculating the efficiency of HMS; second, DEA can effectively avoid the problem of model setting error because of non-parametric estimation; finally, the combination of DEA and bootstrapping truncated regression can overcome the interception problem of efficiency distribution and determine the interactions to improve HMS efficiency (25). Integrating the above analysis, we aimed to put forward scientific suggestions for promoting the high-quality development of HMS in China.

## Materials and methods

### Superefficiency slack-based model-data envelopment analysis

Data envelopment analysis (DEA) is a powerful non-parametric comprehensive evaluation method created by Charnes in 1978 (26). DEA accommodates multiple inputs and outputs in a single measure of efficiency and has become the dominant approach to measuring efficiency in healthcare (27). Additionally, researchers have improved the model in various fields. However, these models have a commonly fatal weakness in that measures are still radial and angular. To solve the effect of slackness caused by input and output, Tone proposed a non-radial slack-based measure of efficiency in data envelopment analysis (SBM-DEA) (28), which was more suitable for evaluating samples with fuzzy interconnect inputs, and further provided target improvement values for the input and output of each inefficient decision-making unit (DMU) (29). Moreover, the traditional DEA model fails to distinguish the DMUs with an efficiency value equal to the highest value “1” (30). As a solution, Xue proposed the superefficiency DEA (SE-DEA) mode in 2002 (31), in which the basic idea was to remove the effective evaluation unit from the set and reevaluate for further evaluation and ranking of multiple DMUs more accurately (32). SE-SBM-DEA is a combination of superefficiency DEA and the SBM model, which addresses excessive input as well as a shortage in output and uses additive models to provide a scalar measure of all inefficiencies (33). Therefore, it has superiority over the traditional DEA model. We used the following description.


(1) $$\rho *=min\frac{1-\frac{1}{N}\sum_{n=1}^{N}\frac{S_{n}^{x}}{x_{kn}^{t}}}{1+\frac{1}{M}\sum_{m=1}^{M}\frac{s_{m}^{y}}{y_{km}^{t}}}$$

In formula (1), ρ ^*^ is the super efficiency value of DMU, $\left(x_{kn}^{t},y_{km}^{t}\right)$ represents the input–output value of the production unit during the period, and $\left(s_{n}^{x},s_{m}^{y}\right)$ represents the relaxation vector of the input–output. This shows that the input is excessive, and the expected output is insufficient when $\left(s_{n}^{x},s_{m}^{y}\right)\geq$0.

### DEA-based malmquist total factor productivity index

In terms of efficiency evaluation, SE-SBM-DEA is mainly used to evaluate the relative performance of medical institutions at all levels in a certain period of time, which cannot continuously compare different periods (34, 35). It is also important to investigate the dynamic development of efficiency for medical institutions at all levels over a while. MTFP can be used in DEA theory to observe the TFP between two periods, objectively reflecting the changes in productivity (36). Caves DW first introduced the MTFP based on Malmquist (37), which has been frequently applied to measure the dynamic efficiency change of DMUs in different periods (38). Färe further decomposed the MTFP into efficiency change (EFFCH), pure efficiency improvement (PECH), technological progress change (TECHCH), and scale efficiency improvement (SECH) as follows, which developed it into a production technology index using a distance function to describe multiple input and output variables (39, 40). The expression of the Malmquist index is expressed using the following formula:


(2) $$\begin{array}{c} \begin{array}{c} MTFP_{i}(x^{t+1},y^{t+1},x^{t},y^{t})=\frac{D_{C}^{t+1}(x^{t+1},y^{t+1})}{D_{C}^{t}(x^{t},y^{t})}\\ \cdot {\left[\frac{D_{C}^{t}(x^{t},y^{t})}{D_{C}^{t+1}(x^{t},y^{t})}\cdot \frac{D_{C}^{t}(x^{t+1},y^{t+1})}{D_{C}^{t+1}(x^{t+1},y^{t+1})}\right]}^{1/2}\\ =EFFCH\cdot TECHCH\\ \begin{array}{c} \begin{array}{c} =\frac{D_{V}^{t+1}(x^{t+1},y^{t+1})}{D_{V}^{t}(x^{t},y^{t})}\cdot \frac{D_{C}^{t+1}(x^{t+1},y^{t+1})/D_{V}^{t+1}(x^{t+1},y^{t+1})}{D_{C}^{t}(x^{t},y^{t})/D_{V}^{t}(x^{t},y^{t})}\\ \cdot {\left[\frac{D_{C}^{t}(x^{t},y^{t})}{D_{C}^{t+1}(x^{t},y^{t})}\cdot \frac{D_{C}^{t}(x^{t+1},y^{t+1})}{D_{C}^{t+1}(x^{t+1},y^{t+1})}\right]}^{1/2}\\ \;=PECH\cdot SECH\cdot TECHCH \end{array} \end{array} \end{array} \end{array}$$

In formula (2), *MTFP*_*i*_ (*TFP*) measures the productivity change of the *i*th DMU between the two periods *t* and *t*+1, and D represents the Shephard distance functions. *x*^*t*^ and *x*^*t*+1^ denote the input vector in period *t*, and *t*+1, *y*^*t*^, and *y*^*t*+1^ denote the output vector.

### Bootstrapping truncated regression

The DEA model measured relative efficiency by comparing and evaluating multi-input and multioutput DMUs, but it did not capture the key factors affecting efficiency. Thus, having calculated the efficiency score, we regressed environmental and managerial factors as the explanatory variables on the OTE to investigate the reasons for inefficiencies in medical institutions at various levels (41). In our study, we took into account the independence, availability, and comprehensiveness of data with the research focus. Therefore, the environmental factors that we considered were based on the literature and included the urbanization rate (UR) and GDP per capita (GDB). Medical technical personnel and beds per 1,000 population (MTPP and BP), the ratio of outpatients to inpatients (OI), the average length of stay (ALoS), and the ratio of doctors to nurses (DN) were determined to be the major managerial factors affecting service efficiency (42). Based on the imbalance in the allocation of healthcare resources existed between urban and rural areas in China (43), the population urbanization reflecting the transition of rural population to urban population might influence residents' decision to seek medical treatment. Like the UR, the GDB could well reflect the local economic development and residents' living standards, which could encourage the choice of high-quality healthcare. The ALoS was a comprehensive index for estimating hospital efficiency, medical quality, and technical level (44). Reducing it can not only minimize the cost of medical resources but also save the cost of medical treatment for patients and increase health production efficiency. The MTPP and BP showed the annual allocation of medical resources in China. At the same time, the DN was an important indicator of the quality of medical institutions, which related to health-related sustainable development. The number of outpatients to the inpatients in the same period represented the functional orientation of disease diagnosis and treatment in medical institutions at various levels and also a strategy to promote public hospitals' participation in HMS.

As the dependent variable, the OTE obtained from SE-SBM-DEA was non-negative; if traditional regression methods such as the ordinary least squares or typical Tobit regression were used directly (45), it would bring serious bias and inconsistency to the parameter estimation due to the existence of a correlation between the measures of efficiency and the error terms (46, 47). To avoid the dependency problem, following Simar and Wilson (48), we adopted bootstrapping truncated regression to explore the factors affecting the OTE of HMS (49) as follows:


(3) $$\begin{array}{l} \begin{array}{c} y_{it}=\alpha_{it}+{\sum^{\text{​}}}_{j=1}^{n}\beta_{j}Z_{j}+\varepsilon_{j}\geq 0;j=1,\ldots \ldots , \end{array}\\ \;N\text{ and}\;\varepsilon_{j}\to N(0,\sigma^{2}) \end{array}$$

Among them, *y*_*it*_ stands for the comprehensive technical efficiency of *i*th DMU in period *t (OTE*_*it*_*)*, *Z*_*j*_ is the set of explanatory variables for j = 1, …, 5, and ε_*j*_ is the error term.

### Variables and data

From 2010 to 2019, we used panel data from secondary and tertiary hospitals and PHCs, including community healthcare centers/stations, township hospitals, and village clinics. Affected by the epidemic situation of COVID-19 in 2020, the efficiency of medical institutions at various levels decreased so significantly that the data were not included in the study. The variables selected for this study, summarized in Table 1, were informed by evidence from similar studies and the availability of data. The correlation for input–output variables was tested by IBM SPSS Statistics 20 and is shown in Table 2. The efficiency results were obtained on a Maxdea ultra 8. Annual percent change (APC) and average annual percent change (AAPC) were used to represent the trend change of the rate. *APC* = (*e*^ρ^−1) × 100%, ρ was the regression coefficient obtained using the Joinpoint Version 4.9.0.1. STATA 16 was used to perform the bootstrapping truncated regression analysis.

**Table 1:** Study variables and data.

**Category**	**Variables**	**Definition**	**References**
***Inputs***	Medical Technical Personnels (MTP, persons)	Doctors with authorization, registered nurses, pharmacists, laboratory physicians, radiologists, and other medical professionals.	Thorsen M (50),Valdmanis V (51), Obure CD (52), Lin R (53)
	Beds (BD, units)	The available beds in medical institutions, excluding for observation, extra beds, etc.	Piubello Orsini L (54), Darabi N (55), Leleu H (56),Mohanta KK (57)
***Outputs***	Outpatients (OP, million)	The total number of outpatients during the financial year.	Yaya S (58), Kazley AS (59), Li SK (60), Linna M (61)
	Inpatients (IP, ten thousand)	The total number of inpatients during the financial year.	Vanková I (62), Otay I (63), Mitropoulos P (64), Cinaroglu S (65)
	The utilization rate of beds (BUR, %)	The number of beds occupied divided by the number of beds open	Stefko R (66), Hu H (67), Ilgün G (68),Gok MS (69)
***Predictors***	Urbanization rate (UR, %)	The proportion of Urban Population at Year-end	Yan C (70), van Noort O (71), Kreng VB (72), Thorsen ML (73)
	GDP per capita (GDB, yuan)	GDP per capita refers to the total output divided by the population	Wang X (74), Halkos GE (75), van Gool K (76), Audibert M (77)
	The average length of stay (ALoS, day)	The total annual number of inpatient days spent/total annual number of admissions	Sarabi Asiabar A (78), Kirigia JM (79), Ayiko R (80)
	Doctors to nurses (DN)	The ratio of doctors to nurses	Kakemam E (81), Jing R (82)
	Medical technical personnel per 1000 population (MTPP)	Number of health technicians divided by population	Ferreira DC (83), Chen A (84)
	Beds per 1000 population (BP)	Number of beds in health facilities divided by population	Top M (85), Hsu Y (86)
	Outpatients to inpatients (OI)	The number of outpatients to the inpatients in the same period	Chowdhury H (87)

**Table 2:** Correlation coefficient analysis of variables.

**Variables**	**MTP**	**BD**	**OP**	**IP**	**BUR**
**MTP**	1	0.877**	0.692**	0.856**	0.422**
**BD**		1	0.284	0.991**	0.661**
**OP**			1	0.247	−0.226
**IP**				1	0.704**
**BUR**					1

** Was significant at the 1% level.

## Results

### Static technical efficiency

**Figure 1:**
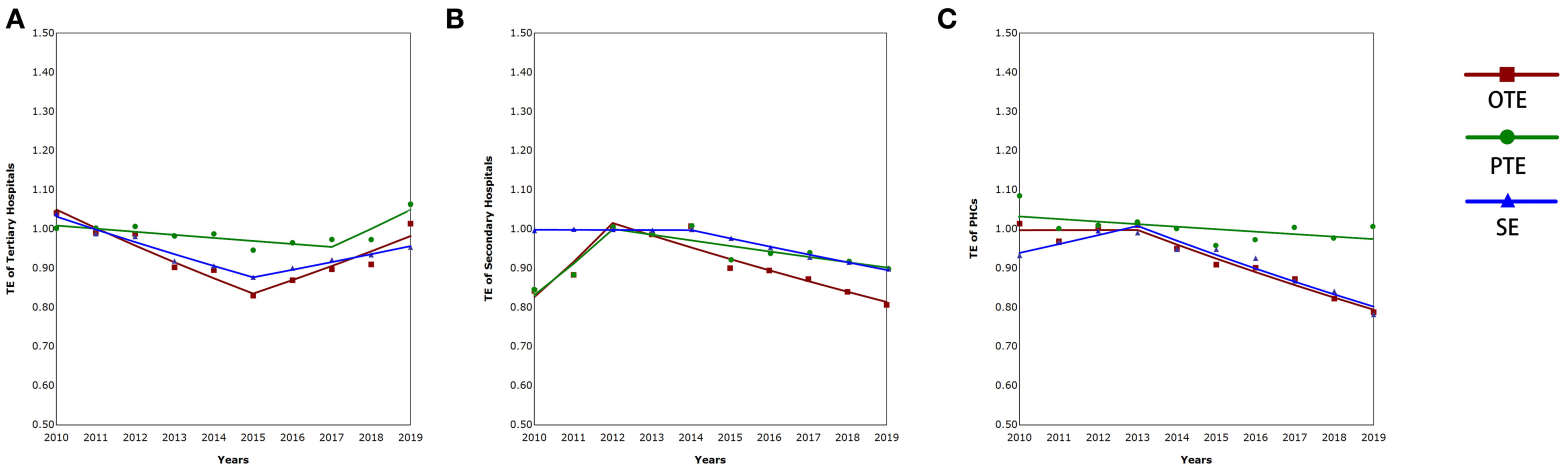
The technical efficiency of tertiary hospitals **(A)**, secondary hospitals **(B)**, and PHCs **(C)**.

From the descriptive statistics trend from 2010 to 2019 (Table 3), we revealed that both input and output in HMS were enhanced annually. Additionally, superior hospitals were more obvious. On average, the results in Table 4, Figure 1 show that the overall technical efficiency (OTE) of tertiary hospitals was 0.93, and it first decreased (APC = −4.46%, *P* < 0.05, in 2010–2015) and then increased acceleratingly (APC = 4.13%, *P* < 0.05, in 2015–2019). The secondary hospitals' OTE was 0.90; however, it first increased (APC = 10.82%, *P* < 0.05, in 2010–2012) and then decreased (APC = −3.11%, *P* < 0.05, in 2012–2019), which was in line with Jiang's results that there was a relatively low OTE of the postreform significantly less than that of the prereform for public county hospitals mainly belonging to secondary hospitals (88). The PHCs generally showed a fluctuating downward trend (APC = −3.73%, *P* < 0.05, in 2013–2019; AAPC = −2.50%, *P* < 0.05, mean equals 0.92 in 2010–2019), a conclusion consistent with previous findings in PHCs (89, 90). Although the “New Medical Reform” began in 2009, the secondary hospitals and PHCs were technically inefficient after the official implementation of the HMS.

**Table 3:** Descriptive statistics of inputs, outputs, and quality attributes, 2010–2019.

	**Medical institutions at various levels**														
	**Tertiary hospitals**					**Secondary hospitals**					**PHCs**				
	**MTP**	**BD**	**OP**	**IP**	**BUR**	**MTP**	**BD**	**OP**	**IP**	**BUR**	**MTP**	**BD**	**OP**	**IP**	**BUR**
**Mean**	2120489.6	1927315.3	141452.28	6596.79	100.75	2008818.7	2131557.4	115019.39	6980.54	86.09	2296494.3	1408035.7	422892.85	4169.6	59.2
**Std. Dev**	646193.92	570900.62	42027.15	2410.45	2.78	272395.78	363515.1	13323.75	1103.48	3.1	324024.05	143122.59	29746.74	207.9	1.69
**Min**	1165082	1065047	76046.3	3096.8	97.5	1646283	1601407	93120.4	5115.7	81.6	1913948	1192242	361155.64	3774.67	56.3
**Max**	3097886	2777932	205701.2	10482.7	104.5	2425917	2665974	134342.5	8380.1	90.7	2920999	1631132	453087.06	4450.05	61.9
**AAPC(%)**	11.39*	11.09*	11.57*	14.41*	−0.84*	4.56*	5.85*	4.17*	5.76*	−0.8	4.75*	3.39*	2.42*	1.16*	−0.04
**P**	<0.001	<0.001	<0.001	<0.001	<0.001	<0.001	<0.001	<0.001	<0.001	0.075	<0.001	<0.001	<0.001	0.027	0.951

*Indicates that the AAPC is significantly different from zero at the alpha = 0.05 level.

**Table 4:** The overall efficiency scores of medical institutions at various levels.

**Years**	**Medical institutions at various levels**											
	**Tertiary hospitals**				**Secondary hospitals**				**PHCs**			
	**OTE**	**PTE**	**SE**	**RTS**	**OTE**	**PTE**	**SE**	**RTS**	**OTE**	**PTE**	**SE**	**RTS**
**2010**	1.0412	1.0017	1.0394	IRS	0.8422	0.8453	0.9963	IRS	1.0139	1.087	0.9327	IRS
**2011**	0.9909	1.0027	0.9883	DRS	0.8833	0.8834	0.9999	IRS	0.9689	1.0024	0.9665	IRS
**2012**	0.988	1.0069	0.9812	DRS	1.005	1.0058	0.9993	IRS	1.0063	1.0107	0.9957	IRS
**2013**	0.9025	0.9825	0.9187	DRS	0.9865	0.9891	0.9974	DRS	1.0101	1.0194	0.9909	DRS
**2014**	0.8953	0.9878	0.9063	DRS	1.0075	1.0084	0.9991	DRS	0.9504	1.0021	0.9484	DRS
**2015**	0.8301	0.9463	0.8772	DRS	0.9004	0.9217	0.9768	DRS	0.9092	0.9586	0.9485	DRS
**2016**	0.8695	0.9652	0.9008	DRS	0.8943	0.9381	0.9533	DRS	0.9013	0.9735	0.9258	DRS
**2017**	0.8975	0.9735	0.9218	DRS	0.8724	0.9398	0.9284	DRS	0.8725	1.0053	0.8679	DRS
**2018**	0.9098	0.9733	0.9347	DRS	0.8400	0.9177	0.9154	DRS	0.8226	0.9776	0.8414	DRS
**2019**	1.0140	1.0638	0.9532	DRS	0.8066	0.8981	0.8981	DRS	0.7878	1.0076	0.7818	DRS
**Mean**	0.93388	0.99037	0.94216		0.90382	0.93474	0.9664		0.9243	1.00442	0.91996	
**APC(%)**	−4.46* (2010–2015, *P* = 0.003); 4.13* (2015–2019, *P* = 0.019)	−0.79* (2010–2017, *P* = 0.028); 4.84(2017–2019, *P* = 0.06)	−3.21* (2010–2015, *P < * 0.001); 2.20* (2015–2019, *P* = 0.009)		10.82* (2010–2012, *P* = 0.036); −3.11* (2012–2019, *P* = 0.001)	9.66* (2010–2012, *P* = 0.043); −1.48* (2012–2019, *P* = 0.023)	−0.02 (2010–2014, P = 0.88); −2.15* (2014–2019, *P < * 0.001)		0.01 (2010–2013, *P* = 0.993); −3.73* (2013–2019, *P* = 0.001)	−0.64 (2010–2019, *P* = 0.081)	2.38 (2010–2013, P = 0.182); −3.74* (2013–2019, *P* = 0.001)	
**AAPC(%)**	−0.73(2010–2019, *P* = 0.298)	0.43(2010–2019, *P* = 0.366)	−0.84* (2010–2019, *P* = 0.007)		−0.17(2010–2019, *P* = 0.844)	0.9(2010–2019, P = 0.288)	−1.21* (2010–2019, *P < * 0.001)		−2.50* (2010–2019, *P < * 0.001)		−1.74* (2010–2019, *P* = 0.004)	

*Indicates that the APC or AAPC is significantly different from zero at the alpha = 0.05 level.

For any DMU, the technical efficiency was effective if pure technical efficiency (PTE) was more than 1. The results showed that secondary hospitals increased at first (APC = 9.66%, *P* < 0.05, in 2010–2012) and then decreased (APC = −1.48%, *P* < 0.05 in 2012–2019) annually. Additionally, 80% of it was always < 1. The opposite was true of tertiary hospitals, and PHCs fluctuated. Scale efficiency (SE) reflects the increase or decrease in returns to scale, which is affected by input (91). The tertiary hospitals decreased first (APC = −3.21%, *P* < 0.05, in 2010–2015), then increased (APC = 2.20%, *P* < 0.05, in 2015–2019), and showed overall downward trends (AAPC = −0.84%, *P* < 0.05, in 2010–2019). Comparatively speaking, the secondary hospitals (APC = −2.15%, *P* < 0.05, in 2014–2019; AAPC = −1.21%, *P* < 0.05, in 2010–2019) and PHCs (APC = −3.74%, *P* < 0.05, in 2013–2019; AAPC = −1.74, *P* < 0.05, in 2010–2019) exhibited a more pronounced downward trend. Generally speaking, 77% of years of all institutions decreased returns to scale.

### Dynamic efficiency evaluation

TFP>1 indicated that the total factor productivity of DMUs showed an increasing trend from t to t-1. In contrast, TFP < 1 indicated downward and TFP = 1 did not change (92). Between 2010 and 2019, the results revealed a negative trend in productivity, with yearly average Rates of 2.73% (tertiary hospitals), 0.51% (secondary hospitals), and 2.70% (PHCs) (Table 5).

**Table 5:** The overall MTFP of health institutions at various levels.

**Periods**	**Medical institutions at various levels**														
	**Tertiary hospitals**					**Secondary hospitals**					**PHCs**				
	**TFP**	**EFFCH**	**TECHCH**	**PECH**	**SECH**	**TFP**	**EFFCH**	**TECHCH**	**PECH**	**SECH**	**TFP**	**EFFCH**	**TECHCH**	**PECH**	**SECH**
2010–2011	0.9747	0.9831	0.9914	1.0045	0.9787	1.0394	0.9981	1.0414	0.9972	1.0008	0.9692	0.9656	1.0037	0.9532	1.013
2011–2012	0.9604	0.9667	0.9935	0.9996	0.9671	1.0785	1.0072	1.0708	0.9688	1.0397	1.0161	0.9976	1.0185	0.9855	1.0124
2012–2013	0.9271	0.9821	0.944	1.0019	0.9802	0.9943	1.0016	0.9927	0.9766	1.0256	1.0034	1.0031	1.0003	0.9904	1.0128
2013–2014	0.9731	0.9833	0.9896	1.0023	0.9811	0.9953	0.9972	0.9981	0.9742	1.0237	0.9527	0.9982	0.9544	0.9853	1.013
2014–2015	0.9421	1.0103	0.9325	1.0087	1.0016	0.9247	0.9917	0.9324	0.9794	1.0126	0.9641	0.9989	0.9653	0.9895	1.0094
2015–2016	0.9925	0.9992	0.9933	1.0051	0.9941	0.991	0.9931	0.9978	1.3171	0.754	0.9889	0.9979	0.991	0.9883	1.0098
2016–2017	0.9959	1.0072	0.9888	1.0136	0.9937	0.987	0.9939	0.993	1.0063	0.9877	0.9708	0.9957	0.975	0.9929	1.0029
2017–2018	0.9885	1.002	0.9865	1.0378	0.9655	0.9745	1.0031	0.9714	1.0467	0.9584	0.9404	0.9968	0.9434	1.0429	0.9559
2018–2019	1.0004	1.0136	0.987	1.048	0.9672	0.9697	0.9934	0.9761	1.0396	0.9555	0.9514	0.9969	0.9543	0.9979	0.9991
2010–2019	0.9727	0.9942	0.9785	1.0135	0.9810	0.9949	0.9977	0.9971	1.0340	0.9731	0.9730	0.9945	0.9784	0.9918	1.0031

During this time period, the EFFCH indicated a reduced trend at an annual rate of 0.58% (tertiary hospitals) and 0.23% (secondary hospitals), with those in 2009 and 2019 being mainly due to the increasing PECH while pronounced decreasing SECH. Furthermore, PHCs showed an upturn by an annual average rate of 0.31% in SECH, but their PECH declined; finally, the EFFCH experienced an average decrease of 0.82%. The results also revealed technology declines, with 81.48% of the TECHCH < 1, and less than one-fifth of the TFP > 1. Therefore, technological progress and innovation were the main contributing factors for improving productivity.

Consistent with the negative changes in SECH for tertiary and secondary hospitals, a gain in OTE could be achieved through downsizing the scale of operation, including equipment, beds, and medical personnel, especially after 2013. Meanwhile, the findings further confirmed that scale inefficiency was the primary cause of inefficiency in Chinese hospitals (93). Moreover, PTE was the key factor that promoted or restricted the improvement of the OTE of medical institutions at various levels. Thus, similar to tertiary hospitals, continuous technology, and quality improvement were foundational approaches to improving the OTE for PHCs and secondary hospitals.

### The relationship between OTE and external and internal factors

The bootstrapping truncated regression of efficiency on medical institutions at various levels was carried out. The results in Tables 6–13 show that the environmental and managerial factors had different significant effects on the OTE of various institutions. As a robustness check of our results, we estimated the verification model, where the OTE remained as the dependent variable, and the explanatory variables were the residuals obtained from OLS regressions of significant affecting factors on each of the indexes, respectively. In tertiary hospitals, the GDB (β = 1.61E-5; *P* < 0.05) was positively correlated. However, OI had a negative association (β = −0.155; *P* < 0.01). In line with previous studies, the economic level affected patients' medical choice preferences (94, 95). However, the most significant negative impact of OI implied that they should provide high-level specialized inpatient services and treat serious or difficult diseases (96). Similarly, OI had a significant negative effect on secondary hospitals (β = −0.127; *P* < 0.05) but failed to pass the robustness test significantly. On the contrary, MTPP negatively affected PHCs (β = −0.238; *P* < 0.01), while BP had a positive influence (β = 0.53; *P* < 0.01). This analysis might suggest that PHCs failed to attract more patients or give full play to the role of “healthy gatekeepers,” and widespread gaps in the quality of PHCs still exist (97), which were limited by the lack of qualified health service providers (98).

**Table 6:** Results of bootstrapping truncated regression for tertiary hospitals.

**Dependent variable: OTE**	**Model 1**	**Model 2**	**Model 3**	**Model 4**	**Model 5**	**Model 6**	**Model 7**
**UR**	−0.007678 (0.313)					−0.055179 (0.718)	−0.040143* (0.091)
**ALOS**		0.029402 (0.238)				−0.133433 (0.867)	−0.114109 (0.590)
**DN**			1.767841** (0.003)			2.187910 (0.808)	1.121549 (0.441)
**MTPP**				−0.024070 (0.443)		0.499124 (0.756)	0.576152 (0.344)
**BP**					−0.029315 (0.339)	−0.432199 (0.811)	−1.1e+00 (0.191)
**GBD**							0.000016** (0.021)
**OI**							−0.155093*** (0.000)
**cons**	1.359638** (0.001)	0.620746** (0.022)	−0.246271 (0.533)	1.071700*** (0.000)	1.079251*** (0.000)	3.239351 (0.771)	8.318436*** (0.000)

Confidence interval 0.95% in parentheses (lower bound, upper bound). ***, **, and * indicate significance at the 1, 5, and 10% levels, respectively. The number of replications is 200 (for each regression).

**Table 7:** Results of bootstrapping truncated regression for tertiary hospitals: estimations of the residuals from GBD.

**Dependent variable: OTE**	**Model 8**	**Model 9**	**Model 10**	**Model 11**	**Model 12**	**Model 13**	**Model 14**	**Model 15**
**Residuals obtained from GDB on UR**	0.056663*** (0.000)						0.004024 (0.922)	0.018884 (0.091)
**Residuals obtained from GDB on ALOS**		0.050837* (0.084)					−0.001966 (0.983)	−0.028727 (0.590)
**Residuals obtained from GDB on DN**			0.030079 (0.186)				−0.031750 (0.618)	0.030556 (0.441)
**Residuals obtained from GDB on MTPP**				0.027324 (0.310)			−0.073887 (0.317)	−0.046138 (0.344)
**Residuals obtained from GDB on BP**					0.056327*** (0.001)		0.167912 (0.113)	0.115565 (0.191)
**Residuals obtained from GDB on OI**						0.035896 (0.198)	−0.045034 (0.142)	−0.038783*** (0.000)
**GBD**								0.000003** (0.005)
**cons**	0.933876*** (0.000)	0.933876*** (0.000)	0.933875*** (0.000)	0.933875*** (0.000)	0.933875*** (0.000)	0.933875*** (0.000)	0.933875*** (0.000)	1.085948*** (0.000)

Confidence interval 0.95% in parentheses (lower bound, upper bound). ***, **, and * indicate significance at the 1, 5, and 10% levels, respectively. The number of replications is 200 (for each regression).

**Table 8:** Results of bootstrapping truncated regression for tertiary hospitals: estimations of the residuals from OI.

**Dependent variable: OTE**	**Model 15**	**Model 16**	**Model 17**	**Model 18**	**Model 19**	**Model 20**	**Model 21**	**Model 22**
**Residuals obtained from OI on UR**	−0.017299 (0.546)						−0.004649 (0.891)	−0.015406 (0.091)
**Residuals obtained from OI on GDB**		0.039372 (0.150)					0.036435 (0.500)	0.031172** (0.021)
**Residuals obtained from OI on ALOS**			−0.042875 (0.095)				0.003543 (0.952)	0.018688 (0.590)
**Residuals obtained from OI on DN**				−0.030011 (0.171)			0.020305 (0.708)	−0.025387 (0.441)
**Residuals obtained from OI on MTPP**					0.026138 (0.420)		0.111729 (0.341)	0.072798 (0.344)
**Residuals obtained from OI on BP**						−0.011022 (0.789)	−0.125613 (0.110)	−0.089452 (0.191)
**OI**								0.019932** (0.009)
**cons**	0.933875*** (0.000)	0.933875*** (0.000)	0.933875*** (0.000)	0.933875*** (0.000)	0.933875*** (0.000)	0.933875*** (0.000)	0.933875*** (0.000)	0.495561** (0.003)

Confidence interval 0.95% in parentheses (lower bound, upper bound). *** and ** indicate significance at the 1 and 5% levels, respectively. The number of replications is 200 (for each regression).

**Table 9:** Results of bootstrapping truncated regression for secondary hospitals.

**Dependent variable: OTE**	**Model 1**	**Model 2**	**Model 3**	**Model 4**	**Model 5**	**Model 6**	**Model 7**	**Model 8**
**UR**	−0.008169 (0.303)						−0.119771 (0.274)	−0.077260 (0.103)
**GDB**		−0.000002 (0.242)					0.000013 (0.622)	0.000011 (0.270)
**ALOS**			−0.001512 (0.993)				−0.374938 (0.304)	−0.241843 (0.285)
**DN**				0.156753 (0.779)			−0.943669 (0.707)	−0.915109 (0.210)
**MTPP**					−0.033424 (0.244)		−0.287304 (0.589)	0.179912 (0.506)
**BP**						−0.031308 (0.319)	0.405656 (0.643)	−0.290336 (0.636)
**OI**								−0.127281** (0.003)
**cons**	1.356813** (0.003)	1.028400*** (0.000)	0.917384 (0.528)	0.787721** (0.049)	1.095212*** (0.000)	1.059083*** (0.000)	1.1e+01*** (0.001)	1.0e+01*** (0.000)

Confidence interval 0.95% in parentheses (lower bound, upper bound). *** and ** indicate significance at the 1 and 5% levels, respectively. The number of replications is 200 (for each regression).

**Table 10:** Results of bootstrapping truncated regression for secondary hospitals: estimations of the residuals from OI.

**Dependent variable: OTE**	**Model 9**	**Model 10**	**Model 11**	**Model 12**	**Model 13**	**Model 14**	**Model 15**	**Model 16**
**Residuals obtained from OI on UR**	−0.063808*** (0.000)						−0.072590 (0.795)	−0.133696 (0.103)
**Residuals obtained from OI on GDB**		−0.058454** (0.002)					0.112425 (0.628)	0.079295 (0.270)
**Residuals obtained from OI on ALOS**			0.010510 (0.773)				0.021944 (0.821)	0.014149 (0.285)
**Residuals obtained from OI on DN**				−0.036238 (0.170)			0.012467 (0.882)	0.018618 (0.210)
**Residuals obtained from OI on MTPP**					−0.059987** (0.002)		0.207123 (0.812)	0.095967 (0.506)
**Residuals obtained from OI on BP**						−0.062799*** (0.000)	−0.320036 (0.618)	−0.126368 (0.636)
**OI**								0.020546 (0.263)
**cons**	0.903825*** (0.000)	0.903825*** (0.000)	0.903825*** (0.000)	0.903825*** (0.000)	0.903825*** (0.000)	0.903825*** (0.000)	0.903825*** (0.000)	0.562972 (0.059)

Confidence interval 0.95% in parentheses (lower bound, upper bound). *** and ** indicate significance at the 1 and 5% levels, respectively. The number of replications is 200 (for each regression).

**Table 11:** Results of bootstrapping truncated regression for PHCs.

**Dependent variable: OTE**	**Model 1**	**Model 2**	**Model 3**	**Model 4**	**Model 5**	**Model 6**	**Model 7**
**UR**	−0.020709*** (0.000)					−0.008095 (0.923)	−0.044691 (0.218)
**GDB**		−0.000006*** (0.000)				−0.000005 (0.862)	−0.000007 (0.608)
**ALOS**			−0.151855** (0.001)			0.162650 (0.795)	0.021304 (0.941)
**DN**				0.401439*** (0.000)		0.155949 (0.957)	0.441144 (0.148)
**OI**					−0.008194 (0.069)	−0.005832 (0.686)	−0.001326 (0.896)
**MTTP**							−0.237540*** (0.000)
**BP**							0.529517*** (0.000)
**cons**	2.072619*** (0.000)	1.216367*** (0.000)	1.884327*** (0.000)	0.224468* (0.061)	1.755233*** (0.000)	0.911431 (0.927)	1.737249** (0.024)

Confidence interval 0.95% in parentheses (lower bound, upper bound). *** and ** indicate significance at the 1 and 5% levels, respectively. The number of replications is 200 (for each regression).

**Table 12:** Results of bootstrapping truncated regression for PHCs: estimations of the residuals from MTTP.

**Dependent variable: OTE**	**Model 8**	**Model 9**	**Model 10**	**Model 11**	**Model 12**	**Model 13**	**Model 14**	**Model 15**
**Residuals obtained from MTTP on UR**	−0.012658 (0.751)						0.005254 (0.932)	0.016097 (0.218)
**Residuals obtained from MTTP on GDB**		0.004092 (0.888)					0.016419 (0.812)	0.008048 (0.608)
**Residuals obtained from MTTP on ALOS**			−0.041736 (0.162)				0.105302 (0.147)	−0.004811 (0.941)
**Residuals obtained from MTTP on DN**				−0.016173 (0.740)			0.004991 (0.967)	0.011619 (0.148)
**Residuals obtained from MTTP on BP**					−0.019226 (0.708)		−0.059351 (0.638)	−0.035886*** (0.000)
**Residuals obtained from MTTP on OI**						−0.067434*** (0.001)	−0.139283** (0.017)	0.010611 (0.896)
**MTTP**								−0.086601** (0.009)
**cons**	0.924300*** (0.000)	0.924300*** (0.000)	0.924300*** (0.000)	0.924300*** (0.000)	0.924300*** (0.000)	0.924300*** (0.000)	0.924301*** (0.000)	1.420178*** (0.000)

Confidence interval 0.95% in parentheses (lower bound, upper bound). *** and ** indicate significance at the 1 and 5% levels, respectively. The number of replications is 200 (for each regression).

**Table 13:** Results of bootstrapping truncated regression for PHCs: estimations of the residuals from BP.

**Dependent variable: OTE**	**Model 16**	**Model 17**	**Model 18**	**Model 19**	**Model 20**	**Model 21**	**Model 22**	**Model 23**
**Residuals obtained from BP on UR**	0.004271 (0.899)						0.002519 (0.937)	0.008446 (0.218)
**Residuals obtained from BP on GDB**		0.011386 (0.745)					0.023149 (0.819)	0.011166 (0.608)
**Residuals obtained from BP on ALOS**			−0.039869 (0.175)				0.097564 (0.150)	−0.004272 (0.941)
**Residuals obtained from BP on DN**				−0.006998 (0.842)			0.002932 (0.968)	0.007162 (0.148)
**Residuals obtained from BP on MTPP**					0.013697 (0.786)		0.033665 (0.506)	0.016805*** (0.000)
**Residuals obtained from BP on OI**						−0.066099** (0.002)	−0.138447** (0.021)	0.010069 (0.896)
**BP**								−0.091306** (0.005)
**cons**	0.924300*** (0.000)	0.924300*** (0.000)	0.924300*** (0.000)	0.924300*** (0.000)	0.924300*** (0.000)	0.924300*** (0.000)	0.924300*** (0.000)	1.377088*** (0.000)

Confidence interval 0.95% in parentheses (lower bound, upper bound). *** and ** indicate significance at the 1 and 5% levels, respectively. The number of replications is 200 (for each regression).

## Discussion

The “inverted triangle” of health resource allocation in China has been the usual course of medical care-seeking behavior (99–101). By comparing medical institutions at various levels in HMS, we further found that the levels of efficiency presented the same form of an “inverted pyramid.” The regional differences in the allocation efficiency of public health resources (102), coupled with hierarchical heterogeneity in HMS, are of particular concern in China.

To obtain the expected medical model, improving the efficiency of subordinate medical institutions is an issue of paramount importance for health policy to ensure the ability to attract and retain patients, especially for PHCs. Evidence indicates that service capability is the primary factor limiting patients' trust and choice of healthcare (103). To improve the effectiveness of HMS, a more practical health policy on improving the professional level of health technicians and the ability of medical service should be formulated.

Considering patients' freedom to choose any medical institution, the mismatching patient flow in the HMS is the main reason for the low efficiency of subordinate medical institutions, on the contrary, overcrowding in high-level general hospitals. Hence, the HMS would not be successful if the voices of the patients were ignored. To address this complicated problem, it is necessary to guide patients to seek medical treatment based on patient-perceived value for service quality and form a reasonable medical order adapting to HMS.

### Implications for hospital managers and policymakers

In promoting the high-quality development of public hospitals in China, managers should take into account not only the balanced allocation of medical resources but also the efficiency. It is imperative to reasonably adjust the scale and service of tertiary hospitals, further motivating the flow of medical talent and service cooperation between tertiary hospitals and low-level hospitals. In addition, the reform of the profit-making mechanism of public hospitals would contribute to improving an active two-way referral in HMS.

Managers should continue to enhance the capacity of medical services through medical alliances, partner assistance, telemedicine, professional coalitions, and so on for secondary hospitals. Moreover, to improve the service capacity of PHCs, training, retaining, and evaluating capable people; improving mechanisms and working conditions; ensuring salary and benefits (104, 105); strengthening the ranks of general practitioners; establishing performance accountability; and optimizing continuing education and professional development are worth considering (106, 107). In addition, policies to strengthen all medicines supply and family doctor contract services would further ensure the primary diagnosis at PHCs (108).

Furthermore, public education and guidance for HMS should be strengthened to change patient care-seeking behavior to hierarchical medical practice. Where the conditions were available, as a pilot, the government should take a powerful way to promote the HMS that patients seek healthcare according to their disease rather than subjectively. On the contrary, it might help achieve HMS to set different reimbursement strategies for different diseases and medical institutions at various levels.

## Conclusion

The inverted pyramid-like effectiveness of the three-level medical system may seriously aggravate the unbalanced development of HMS in China, which was mainly restricted by the medical service capacity and mismatching patient flow. To improve the imbalance, multi-intervention packages focusing on optimizing the allocation of high-quality medical resources, improving the healthcare quality of subordinate medical institutions, and guiding patients to hierarchical diagnosis and treatment should be developed.

## Strength limitation and future research

This study might have several limitations. First, the indicators of efficiency in medical institutions at various levels were mainly considered a quantity metric rather than a quality indicator. Therefore, the research results cannot fully reflect the efficiency level of medical quality. Second, the PHCs include township health centers and village clinics in rural areas, and community health centers (stations) and subdistrict health centers in urban areas. Strictly speaking, the environmental effects on various institutions should be different. Nevertheless, to the best of our knowledge, this study is the first to present levels, trends, and determinants of the effectiveness of the HMS in the past 10 years of China's new medical reform, especially during the phase of the official implementation of HMS since 2015, and then, we put forward targeted countermeasures and suggestions on promoting the high-quality development of HMS.

## Data availability statement

Publicly available datasets were analyzed in this study. This data can be found here: China Health Statistical Yearbooks published between 2010 and 2020.

## Author contributions

YH provided the study idea and drafted the manuscript. WT provided comments and suggestions in revisions of the manuscript. SH gathered and analyzed the data. WL was the corresponding author and the lead writer. All the authors contributed to the revision process and approved the final manuscript.

## Conflict of interest

The authors declare that the research was conducted in the absence of any commercial or financial relationships that could be construed as a potential conflict of interest.

## Publisher's note

All claims expressed in this article are solely those of the authors and do not necessarily represent those of their affiliated organizations, or those of the publisher, the editors and the reviewers. Any product that may be evaluated in this article, or claim that may be made by its manufacturer, is not guaranteed or endorsed by the publisher.
